# A novel respiratory surrogate system with table motion correction and visual feedback for computed tomography

**DOI:** 10.1016/j.phro.2025.100836

**Published:** 2025-09-19

**Authors:** Niklas Andre Lackner, Janis Langkrär, Andre Karius, Oliver J. Ott, Rainer Fietkau, Christoph Bert, Juliane Szkitsak

**Affiliations:** a Department of Radiation Oncology, Universitätsklinikum Erlangen, Friedrich-Alexander-Universität Erlangen-Nürnberg (FAU), 91054 Erlangen, Germany; b Comprehensive Cancer Center Erlangen-EMN (CCC ER-EMN), Erlangen, Germany; c Comprehensive Cancer Center Alliance WERA (CCC WERA), Erlangen, Germany; d Bavarian Cancer Research Center (BZKF), Erlangen, Germany

**Keywords:** Respiratory motion, Novel feedback system, DIBH, 4DCT, Visual feedback, Table sag

## Abstract

**Background and purpose:**

Managing respiratory motion is crucial for computed tomography (CT) in radiotherapy. Clinical surrogate systems and visual coaching can exhibit inaccuracies due to table motion or sag, limiting effectiveness in deep inspiration breath-hold (DIBH) CT and four-dimensional CT (4DCT). This study evaluated a motion-corrected novel surrogate and feedback system to improve DIBH and 4DCT breathing quality.

**Materials and methods:**

The feedback system was evaluated in phantom tests for table sag compensation under incremental loads (up to 104 kg). For patients, DIBH plateau stability metrics (n = 2) and baseline shifts in 4DCT (n = 3) were assessed. In a volunteer study (n = 10), audio and visual feedback were compared in DIBH and 4DCT scenarios, assessing breath-hold stability and breathing regularity, respectively.

**Results:**

In phantom measurements, the impact of table sag on the breathing signal was effectively reduced at maximum load, with baseline shifts limited to −0.2 mm, compared to up to −5 mm in clinical systems. In patients, the system improved DIBH signal stability, as reflected in the plateau drift (−0.2 mm/s vs. −0.8 mm/s), and substantially reduced baseline shifts in 4DCT (−0.2 ± 0.2 mm) when compared to clinical systems (−1.7 ± 0.3 mm). Volunteer tests demonstrated improved DIBH reproducibility with visual feedback (standard deviation: 0.5 mm vs. 1.1 mm with audio feedback). In 4DCT scenarios, visual feedback unified irregular breathers but offered no consistent improvement over audio guidance.

**Conclusion:**

The novel system compensated for table motion in phantom, patient, and volunteer measurements. In feedback scenarios, it performed well in DIBH, while its performance in 4DCT requires further optimization.

## Highlights

• Novel surrogate system improved respiratory motion tracking in computed tomography.
• Compensating table sag in phantom measurements from −5 to −0.2 mm at high loads.
• Improved stability in breath-hold patient scans from −0.8 to −0.2 mm/s.
• Baseline shifts four-dimensional patient scans reduced from −1.7 to −0.2 mm.
• Volunteer study showed visual feedback improved breath-hold metrics.

## Graphical abstract

**Figure:**
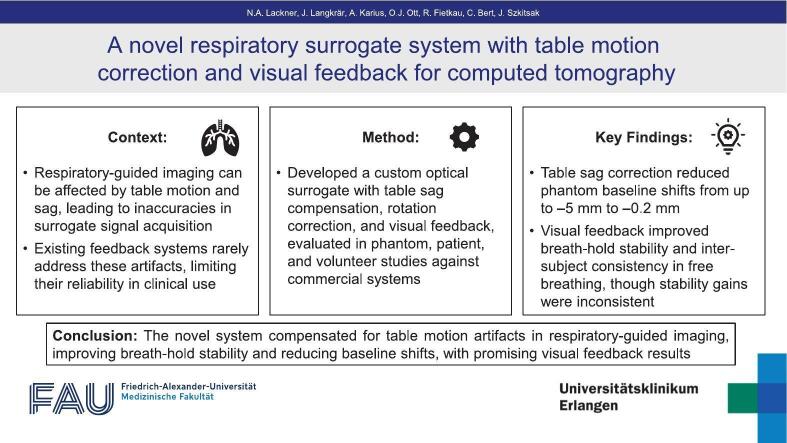

## 1 Introduction

Accurate imaging in radiation therapy is critical for targeting tumors while sparing healthy tissues. Deep inspiration breath-hold (DIBH) and four-dimensional computed tomography (4DCT) are widely used to reduce the impact of respiratory motion on image quality and treatment accuracy [1,2]. DIBH can reduce cardiac dose by increasing the heart-chest wall distance [[3], [4], [5]], while 4DCT captures tumor motion across respiratory phases, enabling optimized treatment planning [6,7].

Both techniques rely heavily on respiratory surrogate systems that synchronize image acquisition with patient breathing or guide patients toward consistent breath-hold levels. Visual feedback has been shown to improve breathing quality and reduce motion artifacts [8,9]. However, a major, yet often overlooked, limitation of current surrogate systems is their inability to distinguish true respiratory motion from signal disturbances induced by CT table movements. Table feed or sagging under patient weight can affect the measured breathing signal [10,11].

In amplitude-based 4DCT binning, even small distortions of a few millimeters can lead to misassigned respiratory phases and image artifacts [12,13]. In visual feedback systems, such distortions may affect the displayed breathing signal, potentially degrading breathing quality if followed by the patient [14]. Despite these potential clinical consequences, table motion artifacts remain underreported and are not routinely corrected in commercial respiratory systems.

In this study, we present a novel in-house developed respiratory surrogate system that combines visual feedback with a correction strategy for CT table motion and sag, using a couch-mounted reference marker. We evaluate its performance in phantom studies, patient measurements, and volunteer experiments, evaluating its impact in both DIBH and 4DCT scenarios.

## 2 Material and methods

### 2.1 CT parameters and protocols

All phantom and patient scans were performed on a SOMATOM go.Open Pro scanner (Siemens Healthineers AG, Germany). 3D DIBH scans were acquired in helical mode using 120 kV and a pitch of 0.8. 4DCT scans were performed in axial mode (Direct i4D, Siemens Healthineers AG, Germany, [15]) with 120 kV and a table feed of 34.5 mm.

### 2.2 Clinical surrogate systems

Two commercial motion monitoring systems were used: Respiratory Gating for Scanners (RGSC, Varian Medical Systems, USA) and SimRT (Vision RT Ltd., UK). RGSC tracks a reflective infrared (IR) marker block using a table-mounted camera to derive a one-dimensional breathing signal [9,12]. SimRT projects structured light on to the patient surface and uses stereo photogrammetry to reconstruct a 3D surface [13,16]. A square region of interest (5 × 5 cm^2^ “patch”) is tracked to derive the breathing signal.

### 2.3 Novel surrogate and feedback system

**Fig. 1:**
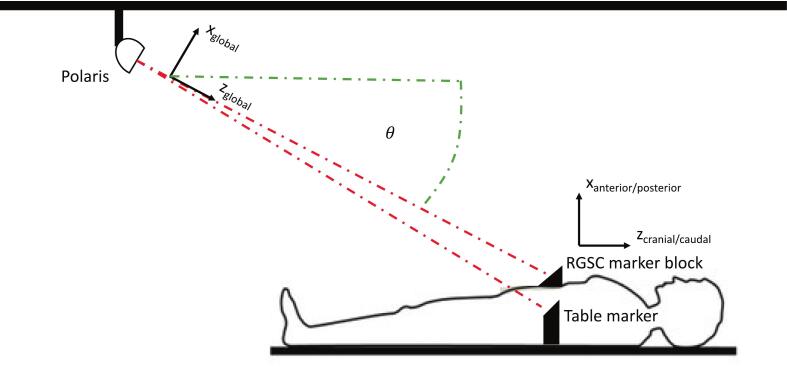
Schematic setup for tracking respiratory and table motion. The ceiling-mounted camera (Polaris) detects the positions of two infrared markers: one placed on the patient (or phantom; RGSC marker block) for respiratory tracking and one mounted on the CT table (table marker). Due to the camera’s angled position, a rotation correction is applied to transform global coordinates into the patient coordinate system, allowing the extraction of anterior–posterior respiratory motion.

Our custom surrogate system uses an optical setup (Polaris Spectra, Northern Digital Inc., Canada) to capture respiratory motion and compensate for CT table movement simultaneously. The system offers sub-millimeter tracking accuracy (0.06 mm root mean square error) and low latency (16.6 ± 1 ms) [17]. A standard RGSC marker block (for the patient or phantom) and a custom-built table marker are placed at the same longitudinal position (Fig. 1). Per-frame 4 × 4 transformation matrices from the Polaris API describe each marker’s position in the global coordinate system. A y-axis pitch rotation corrects for the camera’s angled view, converting global to patient-specific anterior–posterior motion

(1) $$\left[\begin{array}{ccc} cos(\theta ) & 0 & \sin\left(\theta \right)\\ 0 & 1 & 0\\ -\sin\left(\theta \right) & 0 & \cos\left(\theta \right) \end{array}\right]\cdot \left[\begin{array}{c} x_{\mathrm{global}}\\ y_{\mathrm{global}}\\ z_{\mathrm{global}} \end{array}\right]=\left[\begin{array}{c} x_{\mathrm{anterior}/posterior}\\ y_{\mathrm{medial}/lateral}\\ z_{\mathrm{cranial}/caudal} \end{array}\right]$$

The pitch angle θ is extracted from the rotation matrix *R* provided by Polaris (angular error < 0.1°, [18]) using

(2) $$\theta =arcsin\left(-R_{31}\right)$$

where $R_{31}$ is the (3,1) element of the marker’s orientation matrix. The anterior-posterior translations for the patient and the table are computed as

(3) $$x_{\mathrm{breathing}}=x_{\mathrm{global},patient}\cdot cos\left(\theta \right)+z_{\mathrm{global},patient}\cdot sin\left(\theta \right)\;$$

(4) $$x_{\mathrm{table}}=x_{\mathrm{global},table}\cdot cos\left(\theta \right)+z_{\mathrm{global},table}\cdot sin\left(\theta \right)$$

The corrected breathing motion is then derived by subtraction

(5) $$x_{\mathrm{corrected}}=x_{\mathrm{breathing}}-x_{\mathrm{table}}$$

**Fig. 2:**
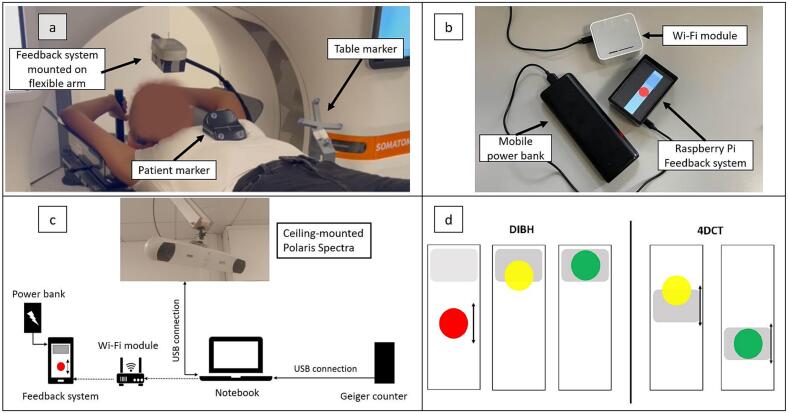
(a) Volunteer setup showing the feedback system mounted on the flexible arm, the patient marker (RGSC marker block), and the 3D-printed table marker. (b) Wi-Fi module for signal transmission, the mobile power bank for power supply, and the Raspberry Pi displaying the visual feedback. (c) System setup: Motion data are transmitted via USB and Wi-Fi for visual patient feedback. The Geiger counter, placed on the CT table near the phantom, enables synchronization of breathing signals with radiation events, with response times on the order of less than 50 ms for both systems. (d) Feedback for the patient indicates whether breathing motion is within the breathing window: green = within range, yellow = at the edge, and red = outside the range. Feedback modes: (1) DIBH mode uses a stationary, draggable breathing window to guide breath-hold positioning. (2) 4DCT mode features a dynamic breathing window provides guidance for regular breathing. (For interpretation of the references to color in this figure legend, the reader is referred to the web version of this article.)

Corrected breathing data is visualized in on a patient-facing display using a compact Raspberry Pi-based feedback system (Fig. 2b). Fig. 2c shows the tracking and feedback system setup. Patients see a ball that moves in sync with their anterior–posterior breathing motion, changing color to indicate whether the motion stays within the intended breathing window, which is shown as a shaded area. Two feedback modes are available: a static breathing window for DIBH and a dynamic breathing window for guiding regular breathing during 4DCT (Fig. 2d).

### 2.4 Data collection and analysis

Breathing signals from RGSC and SimRT included timestamps, amplitudes, and X-ray-on/-off status. The Polaris-based system used a Python 3.7.14 script with the *scikit-surgerynditracker library* [19] to log marker data coupled with X-ray initiation via a GM-10 Geiger counter (Black Cat Systems, USA). For breathing signal comparisons, the curves were aligned based on the first X-ray-on signal. In both phantom and patient measurements, breathing signals from clinical systems were compared to the signal of the Polaris-based surrogate system. For volunteer measurements, only the Polaris-based system was evaluated. In DIBH scans, reproducibility was assessed by comparing mean amplitudes across successive breath-hold plateaus, while stability was quantified through intra-breathhold drifts [20]. In 4DCT scans, baseline shifts were quantified as the difference between the first end-expiration minimum and the last end-expiration minimum (for details, see Lackner et al. [14] or the Supplementary material S.1 and S.2).

### 2.5 Phantom measurements

Measurements were conducted using a CIRS 008A thorax motion phantom (Sun Nuclear, USA). The RGSC marker block was affixed on the motion platform of the phantom and was covered with white tape to ensure detectability across all three systems. A total of nine scans were performed, three for each weight condition (phantom only, +52 kg, +104 kg), to reduce variability and test robustness under varying table sag conditions. To simulate exaggerated table deformation, additional weight was deliberately placed at the head end of the table. Although this configuration does not reflect typical clinical loading, it serves as a controlled worst-case scenario to assess the system’s performance under extreme conditions. Two respiratory patterns were used: (1) sinusoidal breathing followed by a DIBH plateau sequence (breath-hold duration: 6.5 s) for 3DCT in DIBH, and (2) regular sinusoidal breathing (frequency: 0.2 Hz, amplitude: 20 mm) for 4DCT. All scans were performed with consistent start and end positions of the table.

### 2.6 Feasibility for patients

To assess the clinical viability of our surrogate system, respiratory motion measurements were conducted on five patient scans. Two patients underwent the DIBH workflow (DIBH-1,2), and three underwent the 4DCT workflow (4D-1,2,3). During clinical DIBH scans, SimRT provided feedback using the Real Time Coach (Vision RT Ltd., UK) for both training and scan execution, while RGSC and Polaris recorded breathing signals passively. For 4DCT, RGSC was used for the i4D scan mode without visual feedback, while SimRT and Polaris recorded breathing signals passively. All procedures performed were in accordance with the ethical standards of the institutional research committee and with the 1964 Helsinki declaration and its later amendments. Patient consent was not required for this retrospective study per local regulatory policies.

### 2.7 Volunteer measurements

To evaluate our visual feedback system, we recorded respiratory curves from ten volunteers (9 male, 1 female; ages 25–47) while the CT table was moved via remote control between predefined start and end positions, enabling assessment of the system under controlled conditions without ionizing radiation. Although clinical scan sequences were not exactly replicated, table sag typical of a thorax scan was realistically reproduced. Due to the increased table speed compared to clinical scans, compensating for motion was even more demanding, providing a robust test of the system’s performance. All volunteers gave informed consent prior to participation. The study involved only healthy subjects under controlled, non-clinical conditions without ionizing radiation; therefore, approval by an ethics committee was not deemed necessary.

**DIBH:** Each volunteer performed at least three breath-holds (≥5 s each) for each different DIBH scenario: (1) audio feedback without table movement, (2) visual feedback without table movement, and (3) visual feedback with table movement.

**4DCT:** All volunteers breathed for 30 s in each of the three scenarios: (1) normal breathing with audio feedback, no table movement, (2) steady breathing with visual feedback, no table movement, and (3) steady breathing with visual feedback and table movement. In scenarios 2 and 3, visual feedback (Fig. 2d) was used, with the breathing amplitude set to A = 30 mm and cycle duration to T = 10 s, following a cos^6^-function [21]. These parameters were chosen based on a test run with one volunteer and then applied to the entire cohort.

Audio feedback consisted of verbal commands: for DIBH, to inhale deeply and hold their breath for at least 5 s; for 4DCT, to breathe as regularly as possible. Sufficient rest periods were allowed between trials to minimize fatigue effects. In volunteer measurements, statistical analysis was performed on breathing amplitude, breath-hold plateau stability and drift, and breathing cycle duration across feedback scenarios. The Shapiro-Wilk test was used to assess normality. For normally distributed data (p > 0.05), paired t-tests were applied; otherwise, Mann-Whitney U tests were used.

## 3 Results

### 3.1 Phantom measurements

**Fig. 3:**
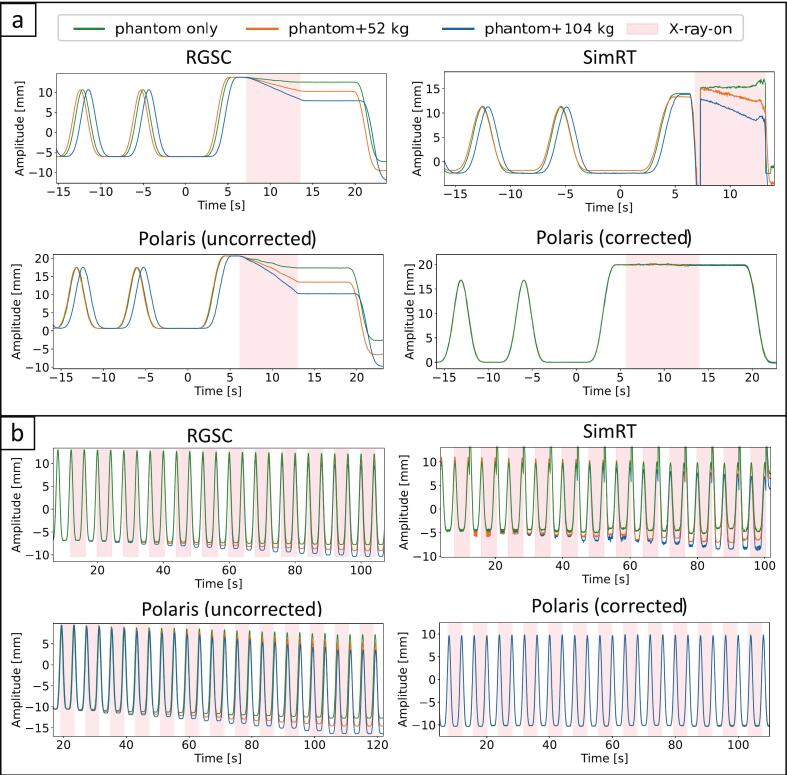
Breathing signals from (a) DIBH and (b) 4DCT phantom experiments comparing commercial systems (RGSC, SimRT) with the in-house Polaris-based surrogate. (a) The top row displays signals from commercial systems; the bottom row shows uncorrected (left) and corrected (right) Polaris-based signals. (b) The top row displays signals from commercial systems; the bottom shows the uncorrected (left) Polaris signal, and the corrected signal (right).

In DIBH scans, RGSC and SimRT both exhibited noticeable baseline shifts with sagging up to −5 mm under the highest load (Fig. 3a, top). For the Polaris-based system, average DIBH amplitudes remained consistent across different table weights (19.9 ± 0.1 mm, mean ± standard deviation (SD) during the X-ray plateau phase) (Fig. 3a, bottom right).

In 4DCT scans, RGSC and SimRT exhibited noticeable baseline shifts due to table sag, sagging up to −4 mm under a load of 104 kg, respectively (Fig. 3b, top), while our Polaris-based system effectively counteracted table sag (Fig. 3b, bottom right), resulting in <0.25 mm table sag across all weight conditions. In both scan modes, SimRT showed artifacts and increased noise because the system accounted for table movement at X-ray transitions by adjusting the patch position to continue deriving breathing data.

### 3.2 Feasibility for patients

For the DIBH scans in patients, breath-hold reproducibility was consistent across all systems, with inter-system differences remaining below 0.5 mm. Although the table was stationary during training, table motion and sagging under the patient’s weight during the actual scan introduced a plateau drift in the recorded breathing signal. During the scan our surrogate system had the most stable plateaus, with signal drift of −0.4 mm/s (patient DIBH-1) and −0.1 mm/s (patient DIBH-2), compared to RGSC’s (−1.1 and −0.6 mm/s) and to SimRT’s (−1.1 and −0.6 mm/s). Fig. 3a shows a DIBH patient breathing signals, with diverging RGSC, SimRT and Polaris breathing signals.

**Fig. 4:**
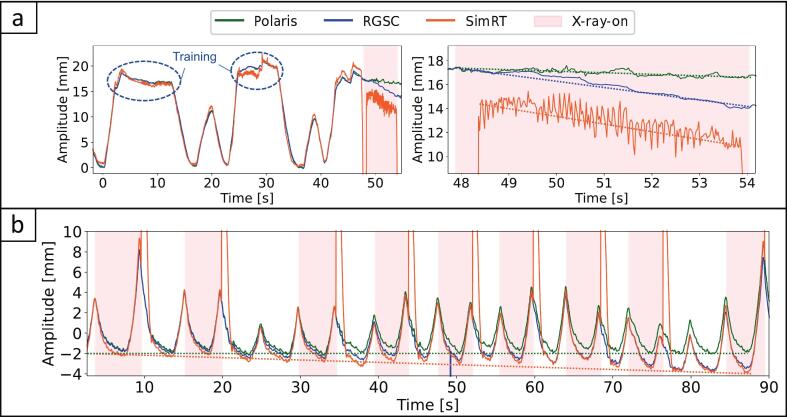
(a) Breathing signals for patient DIBH-2. The left plot shows the full breathing signals, including two training plateaus (as marked), while the right plot provides a zoomed view of the DIBH plateau and the impact of table sag during the scan. SimRT shows a rather noisy curve after X-ray onset, due to patch propagation, which affected breathing signal derivation. (b) Breathing signals for patient 4D-2. The plot illustrates the full breathing signals throughout the 4DCT scan sequence. SimRT showed artifacts at X-ray transitions. Dotted lines indicate drifts and baseline shifts: green for Polaris, blue for RGSC, and orange for SimRT. (For interpretation of the references to color in this figure legend, the reader is referred to the web version of this article.)

For patient 4D-1, Polaris and SimRT had similar baseline shifts with −0.4 and −0.3 mm respectively, while RGSC showed a larger shift of −1.9 mm. For patient 4D-2 (Fig. 4b), RGSC and SimRT had notable baseline shifts, −1.9 mm and −1.6 mm, respectively, whereas Polaris remained stable (−0.3 mm). Patient 4D-3 showed a slight positive trend for Polaris (0.1 mm), contrasting with RGSC and SimRT's shifts (−1.3 and −0.4 mm). In total, for all 4DCT patient acquisitions, Polaris had the lowest average shift (−0.2 ± 0.2 mm), outperforming RGSC (−1.7 ± 0.3 mm) and SimRT (−0.8 ± 0.6 mm).

### 3.3 Volunteer measurements

**DIBH scenario:** Table 1 shows breath-hold plateau reproducibility and stability for the ten volunteers across the three tested scenarios. The mean SD of plateau amplitudes across the ten volunteers decreased from 1.1 mm with audio feedback (scenario 1) to 0.5 mm with visual feedback (scenario 2) and 0.4 mm with visual feedback and table movement (scenario 3).

**Table 1:** Breathing measurements across three scenarios: audio feedback without table movement, visual feedback without table movement, and visual feedback with table movement. Results represent mean values ± SD for each individual volunteer. The second-to-last row reports the mean ± SD across all volunteers; the final row presents the mean of the volunteers’ SDs ± the SD of those SDs. (a) Reproducibility and stability of DIBH measurements, including amplitude and drift across three plateaus for each scenario. (b) Breathing amplitude and breathing duration stability during 4DCT scenarios, including breathing amplitude and cycle duration.

**DIBH**	Audio feedback		Visual feedback		Visual feedback	
	No table movement		No table movement		Table movement	
Volunteer #	Amplitude (mm)	Drift (mm/s)	Amplitude (mm)	Drift (mm/s)	Amplitude (mm)	Drift (mm/s)
1	4.2 ± 0.7	−0.3 ± 0.1	10.9 ± 0.2	−0.2 ± 0.0	14.4 ± 0.4	−0.1 ± 0.2
2	5.0 ± 1.2	−0.5 ± 0.1	10.5 ± 0.2	−0.2 ± 0.1	18.2 ± 0.1	−0.3 ± 0.2
3	23.8 ± 1.3	−0.5 ± 0.4	16.7 ± 0.4	−0.0 ± 0.0	18.2 ± 0.7	−0.2 ± 0.2
4	21.0 ± 0.8	−0.0 ± 0.3	12.7 ± 0.5	−0.5 ± 0.2	17.1 ± 0.2	−0.4 ± 0.0
5	13.1 ± 0.2	−0.4 ± 0.1	12.1 ± 1.0	−0.2 ± 0.0	15.4 ± 0.7	−0.4 ± 0.0
6	13.1 ± 2.8	−0.6 ± 0.1	11.1 ± 0.3	−0.2 ± 0.3	19.3 ± 0.7	−0.4 ± 0.4
7	6.8 ± 0.8	−0.0 ± 0.1	10.3 ± 1.8	−0.4 ± 0.1	6.9 ± 0.2	0.0 ± 0.1
8	17.0 ± 0.7	−1.0 ± 0.1	10.9 ± 0.2	−0.2 ± 0.0	11.6 ± 0.2	−0.1 ± 0.2
9	10.3 ± 0.6	−0.3 ± 0.2	4.8 ± 0.1	0.0 ± 0.1	9.5 ± 0.7	−0.2 ± 0.6
10	11.6 ± 1.6	−0.0 ± 0.1	9.5 ± 0.4	0.1 ± 0.0	18.8 ± 0.3	0.1 ± 0.1
Mean total	12.6 ± 6.5	−0.4 ± 0.3	11.0 ± 2.9	−0.2 ± 0.2	14.9 ± 4.3	−0.2 ± 0.2
Mean SD	1.1 ± 0.7	0.2 ± 0.1	0.5 ± 0.5	0.1 ± 0.1	0.4 ± 0.3	0.2 ± 0.2

**4DCT**	Audio feedback		Visual feedback		Visual feedback	
	No table movement		No table movement		Table movement	
Volunteer #	Amplitude (mm)	Duration (s)	Amplitude (mm)	Duration (s)	Amplitude (mm)	Duration (s)
1	14.4 ± 0.7	4.4 ± 0.2	27.5 ± 3.0	9.7 ± 1.8	30.2 ± 3.6	8.4 ± 2.9
2	8.1 ± 1.3	3.0 ± 0.2	32.4 ± 1.8	9.7 ± 0.7	30.7 ± 2.4	9.9 ± 0.6
3	41.0 ± 2.8	4.5 ± 0.3	32.9 ± 1.3	10.0 ± 0.3	33.6 ± 1.7	10.0 ± 0.2
4	11.7 ± 1.0	5.7 ± 1.6	31.7 ± 2.5	9.9 ± 0.7	30.5 ± 1.6	9.9 ± 0.4
5	8.2 ± 0.9	4.1 ± 0.2	15.1 ± 7.6	6.8 ± 3.0	25.2 ± 1.3	9.9 ± 0.3
6	12.1 ± 1.1	5.3 ± 0.4	30.0 ± 2.1	11.3 ± 1.9	29.0 ± 1.5	9.9 ± 0.3
7	14.7 ± 0.9	7.9 ± 0.2	30.4 ± 2.3	9.9 ± 0.0	29.9 ± 1.6	10.0 ± 0.2
8	14.4 ± 0.4	5.6 ± 0.5	31.9 ± 2.5	9.4 ± 2.2	26.1 ± 2.7	9.3 ± 1.4
9	20.6 ± 1.8	4.8 ± 0.4	31.2 ± 2.3	10.1 ± 1.7	30.8 ± 2.8	9.8 ± 1.0
10	16.9 ± 1.7	9.7 ± 0.9	27.6 ± 2.4	10.3 ± 1.0	35.4 ± 2.4	10.3 ± 0.6
Mean total	16.2 ± 9.5	5.5 ± 2.0	29.1 ± 5.2	9.7 ± 1.1	30.1 ± 3.0	9.7 ± 0.5
Mean SD	1.3 ± 0.7	0.5 ± 0.4	2.8 ± 1.8	1.3 ± 0.9	2.2 ± 0.7	0.8 ± 0.8

Reproducibility improved with visual feedback in scenario 2 (8 of 10 volunteers; p = 0.08) and showed further gains in scenario 3 (p = 0.02), likely reflecting a learning effect.

**Fig. 5:**
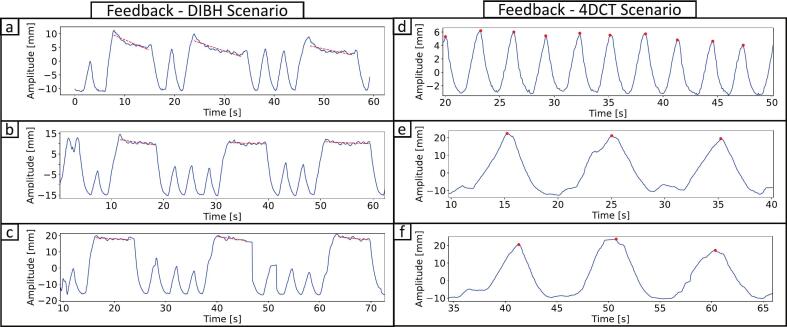
On the left: DIBHs for all scenarios of a volunteer: (a) audio feedback without table movement, (b) visual feedback without table movement, and (c) visual feedback with table movement. In (b) and (c), the novel feedback system displayed amplitude and a DIBH breathing window. Plateau gradients are shown as dashed red lines. On the right: 4DCT scenario of a volunteer for all scenarios: (d) normal breathing with audio feedback and no table movement, (e) steady breathing with visual feedback and no table movement, and (f) steady breathing with visual feedback and table movement. In (e) and (f), visual feedback controlled breathing amplitude (A = 30 mm) and cycle duration (T = 10 s) using a cos^6^-function. Red dots mark breathing maxima, and breathing cycles were segmented by identifying max-to-max intervals. (For interpretation of the references to color in this figure legend, the reader is referred to the web version of this article.)

Furthermore, the analyzed plateau drift yielded mean drift of −0.4 ± 0.3 mm/s (scenario 1), −0.1 ± 0.3 mm/s (scenario 2), and −0.2 ± 0.3 mm/s (scenario 3). Fig. 5, Fig. 5 shows an overall flatter plateau (red dashed line) with visual feedback. Stability improved in 8 of 10 volunteers in scenario 2 and 6 of 10 in scenario 3, compared to scenario 1.

For the DIBH data, all datasets passed the Shapiro-Wilk normality test (p > 0.059). Breathing amplitude was significantly higher with visual feedback and table movement compared to visual feedback without table movement (p < 0.05), while no significant differences were found in amplitude reproducibility between audio and visual feedback (p = 0.48 and p = 0.35), or in breathing drift across any scenario (all p > 0.13), which is positive as it indicates that table motion and sag were effectively compensated.

**4DCT scenario:** Table 1 shows breathing amplitude and cycle length stability for ten volunteers. Amplitude stability had a mean SD of 1.3 mm with audio feedback (scenario 1), increasing to 2.8 mm with visual feedback (scenario 2), and slightly decreasing to 2.2 mm with table movement (scenario 3). These results indicate visual feedback did not improve amplitude stability in 4DCT scenarios compared to the audio feedback, with only 1 of 10 volunteers showing improvement.

Furthermore, breathing cycle duration stability was assessed across volunteers as well. The mean SD increased from 0.6 s with audio feedback (scenario 1) to 1.4 s with visual feedback (scenario 2), then decreased to 0.8 s with visual feedback and table movement (scenario 3), again suggesting a learning effect during the visual feedback from scenario 2 to scenario 3. The feedback system did not substantially enhance duration stability compared to the audio feedback, with only 2 of 10 volunteers improving in scenario 2 and 4 of 10 in scenario 3. Fig. 5, Fig. 5, Fig. 5 shows breathing signals from a volunteer, with more consistent amplitudes and cycle lengths in scenarios 2 and 3. Breathing amplitudes and durations were significantly different between audio feedback and both visual feedback scenarios (p < 0.05, Mann-Whitney U tests), while independent t-tests showed no significant differences between the two visual feedback scenarios for amplitude (p = 0.59) and duration (p = 0.94), indicating that reproducible breathing was achieved.

## 4 Discussion

This study evaluated a novel in-house optical surrogate and feedback system for respiratory motion control in DIBH and 4DCT imaging. Benchmarking against commercial systems in phantom and clinical settings showed improved surrogate signal quality, with greater reproducibility and stability in DIBH breathing signals and reduced baseline shifts from table sag in 4DCT breathing signals. The integrated visual feedback functionality was validated in a volunteer cohort.

A previous study identified susceptibility of commercial surrogate and feedback systems to breathing artifacts from table motion and sag [14]. Building on this, we introduced key improvements: table sag compensation via a dedicated 3D-printed table marker, a ceiling-mounted system with rotation correction, and integrated visual feedback. Notably, this is the first clinical evaluation of a Polaris-based system benchmarked against commercial systems.

Phantom experiments demonstrated robustness under high table loads, with improved signal stability over commercial systems. In DIBH, breathing signal stability was higher; in 4DCT, baseline shifts from table sag were minimized. This supports clinical applicability, especially in heavier patients prone to table deflection.

To test robustness under extreme conditions, phantom scans were performed with artificial loading beyond clinical scenarios. Despite the extreme setup, the system maintained stable performance, confirming the effectiveness of sag correction even under exaggerated sag.

In patient DIBH scans, all systems achieved < 0.5 mm breath-hold level deviation, but during the CT scan the Polaris-based system showed superior plateau stability with lower signal drift (0.2 ± 0.2 mm/s) compared to RGSC (−0.8 ± 0.4 mm/s) and SimRT (−0.9 ± 0.3 mm/s), due to table motion compensation. In patient 4DCT acquisitions, Polaris showed the smallest baseline shifts (−0.2 ± 0.2 mm) compared to RGSC (−1.7 ± 0.3 mm) and SimRT (−0.8 ± 0.6 mm). While drift values as low as 0.2 mm/s approach the resolution limit, it reflects a consistent suppression of drift and displacement by the Polaris-based system, which produced a more robust and artifact-resistant surrogate signal. Reducing table-induced drift is clinically relevant, as uncorrected motion can cause artifacts in visual coaching [22] and in amplitude-based 4DCT reconstruction [23,24].

Patient weight distribution and its potential impact on table sag were analyzed in a previous study [14], involving 29 4DCT and 41 DIBH patients (mean weights: 69 ± 13 kg and 74 ± 14 kg, respectively). Reported baseline sag reached up to −1.7 ± 0.7 mm in 4DCT and DIBH-related table drift up to −5.3 ± 3.7 mm, underscoring the clinical relevance of table deflection correction.

Volunteer DIBH scenarios showed that visual feedback significantly improved breath-hold reproducibility (p < 0.05). Stability improved in 80 % of volunteers without table travel and 60 % with table travel, reflected by reduced SD and less plateau signal drift. In contrast, volunteers having only audio feedback struggled to maintain a flat plateau. Visual feedback enabled more precise breathing adjustments.

In 4DCT volunteer scenarios, individual breathing regularity showed no improvement with visual feedback compared to audio feedback, likely because the volunteers were already breathing regularly. However, visual feedback helped align breathing patterns across volunteers, reducing inter-subject variability. A limitation of this study is the use of a 30 mm amplitude for regular breathing guidance, which was significantly higher (p < 0.05) than the average natural amplitude observed in Scenario 1 (16.2 ± 9.0 mm). This amplitude was selected based on preliminary testing in a single individual who exhibited unusually large breathing amplitudes, which were not representative of the broader volunteer cohort. In retrospect, this highlights the limitations of one-size-fits-all approaches, as individual differences in breathing mechanics and the cos^6^-based model's failure to represent natural respiration in any volunteer limited its applicability. A patient-specific strategy, starting with audio-guided natural breathing, followed by mathematical fitting (e.g., polynomial) and personalized visual feedback, could potentially improve breathing quality in 4DCT.

Several feedback systems have been developed to improve respiratory control in radiotherapy, including video goggle systems for compliance [25,26] and marker-based feedback for breath-hold reproducibility [27]. While visual feedback is common, Kini et al. found audio prompting more effective for stability [28]. However, none of these systems addressed table motion artifacts to improve surrogate signal quality, and to our knowledge, this is the first study to do so.

A limitation is that no feedback was used in 4DCT patient observations. For DIBH patients, a different system was used that does not compensate for table sag. Direct feedback with our system would require medical device compliance, which was beyond this feasibility study’s scope.

Table sag and motion artifacts were explicitly corrected using our custom system; similar strategies could be integrated into commercial workflows. Incorporating direct deflection measurements may improve calibration and system reliability. Neither clinical system, RGSC or SimRT, compensates for sag or accounts for table load during calibration, potentially introducing systematic errors. Our results show such corrections are both feasible and beneficial.

Further limitations include a small, homogeneous, healthy, young volunteer cohort, and simulated table movement instead of real CT scans. Future studies should involve larger, more diverse populations to improve applicability.

In conclusion, the in-house developed surrogate system reliably corrected table motion artifacts and stabilized respiratory signals in CT scans, as demonstrated in phantom and patient breathing signal comparisons. In volunteer tests, the feedback system effectively guided breathing in DIBH and 4DCT scenarios. While further refinement is needed for 4DCT, the system proved effective in DIBH, improving stability and reproducibility while compensating table motion.

## Declaration on Generative AI

Generative AI (OpenAI ChatGPT v-4o) was utilized for proofreading this manuscript. All AI-generated suggestions were carefully reviewed and edited by the authors to ensure accuracy and clarity. The authors take full responsibility for the final content.

## CRediT authorship contribution statement

**Niklas Andre Lackner:** Conceptualization, Methodology, Software, Data curation, Formal analysis, Investigation, Writing – original draft, Writing – review & editing. **Janis Langkrär:** Conceptualization, Methodology, Software, Data curation, Formal analysis, Investigation, Writing – original draft, Writing – review & editing. **Andre Karius:** Writing – review & editing. **Oliver J. Ott:** Writing – review & editing. **Rainer Fietkau:** Writing – review & editing. **Christoph Bert:** Conceptualization, Methodology, Supervision, Writing – review & editing. **Juliane Szkitsak:** Conceptualization, Methodology, Supervision, Writing – review & editing.

## Declaration of competing interest

The authors declare the following financial interests/personal relationships which may be considered as potential competing interests: The Universitätsklinikum Erlangen and the Department of Radiation Oncology have institutional research grants with Siemens Healthineers AG and VisionRT Ltd that are not directly related to the present work.
