# Supplementary material for: A novel respiratory surrogate system with table motion correction and visual feedback for computed tomography
Source: Phys Imaging Radiat Oncol. 2025 Sep 19;36:100836. doi: 10.1016/j.phro.2025.100836 (PMC12495361; doi:10.1016/j.phro.2025.100836)
Supplement: Supplementary Data 1 [file mmc1.pdf]

Supplementary Material

S.1 DIBH metrics

Reproducibility was evaluated by calculating the difference in mean breathing amplitudes over the DIBH plateau between successive DIBH plateaus

$$\Delta y = \text{mean}(\text{DIBH}_{\text{prior}}) - \text{mean}(\text{DIBH}_{\text{current}}), \tag{S.1}$$

where $\text{DIBH}_{\text{prior}}$ and $\text{DIBH}_{\text{current}}$ represent consecutive breath-hold plateaus. Lower values of Δy indicate better reproducibility.

Stability was assessed as the estimated breath-hold drift rate, derived from fitting each DIBH instance with a linear model:

$$y(t) = y_{\text{start}} + m \cdot t, \tag{S.2}$$

where y_{start} is the amplitude at the start of the breath-hold, m represents the drift rate in mm/s, and t is the time elapsed during the DIBH. A low value of m indicates good stability with minimal drift over time. Drift rates m were evaluated for each DIBH instance in the dataset to assess the consistency of breath-hold stability. Reproducibility and stability in DIBH were evaluated using metrics slightly adapted from Cervino et al. [1], as illustrated in Figure S1.

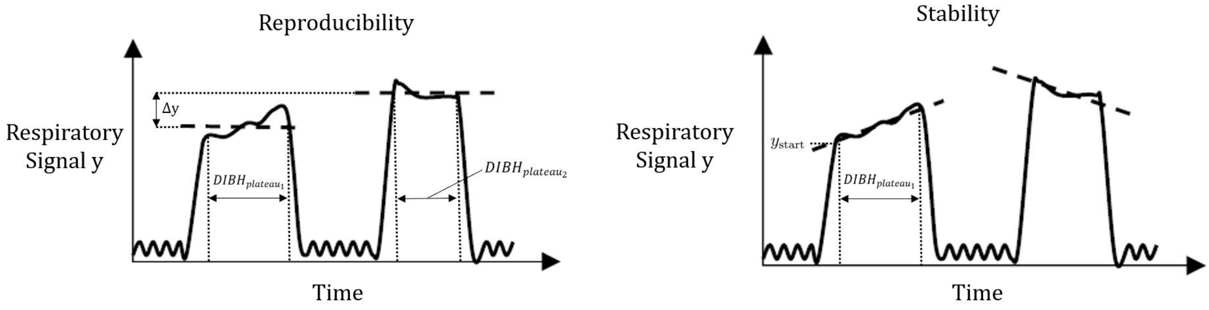

Figure S1. Graphical representation of reproducibility (left) and stability (right) in DIBH. Dashed lines in the left plot indicate the mean amplitude levels of successive DIBH plateaus, while dashed lines in the right plot represent linear fits used to estimate intra-breathhold drift. Figure adapted from Cervino et al. [1].

S.2 4DCT baseline shifts

Baseline shift in a breathing curve refers to a deviation from the initial baseline level of respiration. It was evaluated in phantom and patient 4DCT acquisitions by calculating the difference between the first and last signal minimum values, where the minima correspond to the breathing valleys as illustrated in Figure S2, adapted from Lackner et al. [2].

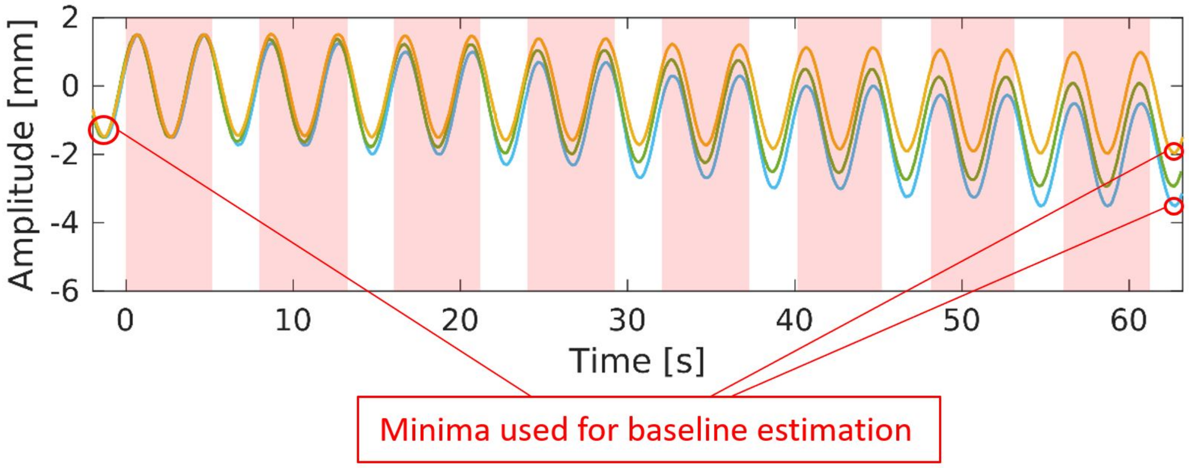

Figure S2. Graphical representation of baseline shift in example breathing curves, evaluated by measuring the difference between the first and last signal minimum values (breathing valleys highlighted by red circles). This figure was adapted from Lackner et al. [2].

References

[1] Laura I. Cerviño, Sonia Gupta, Mary A. Rose, Catheryn Yashar, and Steve B. Jiang. "Using surface imaging and visual coaching to improve the reproducibility and stability of deep-inspiration breath hold for left-breast-cancer radiotherapy". In: *Physics in Medicine and Biology* 54.22 (2009), pp. 6853–6865. DOI: 10.1088/0031-9155/54/22/007.

[2] Niklas Lackner, Lisa Dietrich, Andre Karius, Rainer Fietkau, Christoph Bert, and Juliane Szkitsak. "Investigating the effects of table movement and sag on optical surrogate-driven respiratory-guided computed tomography". In: *Journal of Applied Clinical Medical Physics* 26.2 (Feb. 2025). Epub 2024 Nov 29. PMID: 39611794; PMCID: PMC11799900, e14565. DOI: 10.1002/acm2.14565.
